# Crosslinked Biodegradable Hybrid Hydrogels Based on Poly(ethylene glycol) and Gelatin for Drug Controlled Release

**DOI:** 10.3390/molecules29204952

**Published:** 2024-10-19

**Authors:** Zhenzhen Zhao, Zihao Qin, Tianqing Zhao, Yuanyuan Li, Zhaosheng Hou, Hui Hu, Xiaofang Su, Yanan Gao

**Affiliations:** 1 School of Advanced Agricultural Science, Weifang University, Weifang 261061, China; 20230012@wfu.edu.cn; 2 College of Chemistry, Chemical Engineering and Materials Science, Shandong Normal University, Jinan 250014, China; q1270907106@163.com; 3 Key Laboratory of Ministry of Education for Advanced Materials in Tropical Island Resources, Hainan University, Haikou 570228, China; 22220856000014@hainanu.edu.cn (T.Z.); hhu@hainanu.edu.cn (H.H.); sxf@hainanu.edu.cn (X.S.); ygao@hainanu.edu.cn (Y.G.)

**Keywords:** hydrogel, poly(ethylene glycol), gelatin, degradable, drug release

## Abstract

A series of hybrid hydrogels of poly(ethylene glycol) (PEG) were synthesized using gelatin as a crosslinker and investigated for controlled delivery of the first-generation cephalosporin antibiotic, Cefazedone sodium (CFD). A commercially available 4-arm-PEG–OH was first modified to obtain four-arm-PEG–succinimidyl glutarate (4-arm-PEG–SG), which formed the gelatin–PEG composite hydrogels (S_n_N_m_) through crosslinking with gelatin. To regulate the drug delivery, S_n_N_m_ hydrogels with various solid contents and crosslinking degrees were prepared. The effect of solid contents and crosslinking degrees on the thermal, mechanical, swelling, degradation, and drug release properties of the hydrogels were intensively investigated. The results revealed that increasing the crosslinking degree and solid content of S_n_N_m_ could not only enhance the thermal stability, swelling ratio (SR), and compression resistance capacity of S_n_N_m_ but also prolong the degradation and drug release times. The release kinetics of the S_n_N_m_ hydrogels were found to follow the first-order model, suggesting that the transport rate of CFD within the matrix of hydrogels is proportional to the concentration of the drug where it is located. Specifically, S_1_N_1_-III showed 90% mass loss after 60 h of degradation and a sustained release duration of 72 h. The cytotoxicity assay using the MTT method revealed that cell viability rates of S_1_N_1_ were higher than 95%, indicating excellent cytocompatibility. This study offers new insights and methodologies for the development of hydrogels as biomedical composite materials.

## 1. Introduction

In the past few decades, extensive research has been carried out in the design and preparation of drug delivery systems, such as nanoparticles, micelles, hydrogels, liposomes, porous frame materials, etc. Compared to conventional drug formulations, these new delivery materials show various advantages, including high drug loading efficiency, controllable release rate, and long delivery periods [1,2]. Among them, hydrogels are three-dimensional, crosslinked polymeric networks that are durable to swelling in water and that maintain their shape without obvious deformation. The storage of plenty of water endows hydrogels with good biocompatibility, excellent hydrophilicity, and similar mechanical properties to human soft tissues [3]. Importantly, hydrogels can be formulated into various physical forms. As a consequence, hydrogels have been commonly used in clinical practice and experimental medicine for a large range of applications, such as tissue engineering [4], regenerative medicine [5], biosensors [6], and adhesion barrier materials [7,8,9,10].

Due to their unique physical properties, hydrogels have also attracted much attention in the field of drug delivery. Their pore structure can be adjusted by controlling the density of crosslinks in the matrix, and their chemical properties can easily be tuned by selecting proper polymers and processing techniques. The rich porosity of hydrogels allows the loading of targeted drugs into the gel matrix and subsequent drug release in a controlled manner. By modulating the pore structures and chemical compositions of the hydrogels, the release kinetics and time can be tailored to meet the therapeutic requirements. A prolonged therapeutic effect [11], reduced dosing frequency [12], and improved patient compliance [13,14,15] have been achieved due to the sustained drug release capability of hydrogels. Furthermore, stimuli-responsive hydrogels have been developed by incorporating responsive species into the hydrogel matrix, such as temperature-sensitive or pH-sensitive polymers and light-responsive moieties. Through external stimuli, the selective release of drugs can be triggered, which can minimize systemic side effects and maximize therapeutic efficacy [16,17,18].

Biocompatibility is a critical criterion for assessing the safety of medical hydrogels. Excellent biocompatibility means that a substance will not cause significant immune or tissue rejection reactions, thereby reducing complications and risks during the treatment process [19,20]. Meanwhile, it would be ideal if the hydrogels could be degraded naturally or adsorbed by the body after fulfilling their function [21], which is essential for preventing material removal during secondary surgery and promoting patient recovery. Poly(ethylene glycol) (PEG), which has excellent water-solubility and biocompatibility, exhibits rapid clearance from the body [22,23]. However, PEG hydrogels alone generally cannot provide an ideal environment in clinical practice and experimental medicine. The coupling of proteins or other biological molecules to PEG can endow it with good biological activity, rendering PEG surfaces resistant to cell and protein adsorption. These properties enable PEG-containing hydrogels to be ideal candidates for drug delivery systems. Therefore, synthetic PEG-containing hydrogels have been developed, and they exhibit advantages over natural hydrogels, such as the capability for adjustable mechanical properties and easy control of the scaffold structure and chemical compositions, etc. In this regard, PEG and multi-arm-PEG derivatives with various active end groups are more frequently used to prepare PEG-based hybrid hydrogels for medical applications, such as PEG/albumin hydrogels for loading protein [24,25], PEG/collagen hydrogels for tissue regeneration, and tissue engineering scaffolds [26,27]. As a natural polypeptide polymer, gelatin not only offers wide availability and low cost but also possesses a chemically manipulable structure, which makes it extensively investigated in biomaterials for the design of drug delivery systems and tissue engineering scaffolds [28,29,30]. Although PEG-modified gelatin nanoparticles or microspheres have been developed as long-circulating gene intracellular delivery systems [31,32,33], PEG–gelatin medical hydrogels with satisfactory mechanical properties have scarcely been reported.

The covalent crosslinked gelatin with multi-arm-PEG not only integrates the advantages of both materials but also overcomes their limitations. Crosslinking can enhance the mechanical properties and stability of the composite, prolonging its functional lifespan within the body and improving the efficacy and durability of the material in drug delivery systems. Furthermore, by adjusting the crosslinking degree and solid content, the physicochemical properties of the hybrid hydrogels can be regulated to meet specific requirements for sustainable release in drug delivery systems. In recent years, PEG–gelatin hybrid hydrogels have been developed. For instance, an injectable PEG–gelatin hydrogel was designed from a multifunctional PEG-based hyperbranched polymer and a commercially available thiolated gelatin. Although the PEG–gelatin hydrogel can be used to encapsulate murine adipose-derived stem cells (ASCs), sulfides can undergo an S-phosphonate esterification reaction with proteins, thereby affecting the enzymatic activity and structural stability of the proteins [34]. To improve the mechanical and cell adhesive properties, a series of hybrid hydrogels based on PEG and natural polymer gelatin were also prepared, where PEG dimethacrylate (PEG-dMA) and gelatin methacrylate (GelMA) macromers were first prepared and underwent photo-crosslinking in water in different ratios [35]. However, the operation process of this strategy is complex and cannot be scaled up for preparation. Consequently, the design and preparation of PEG–gelatin hydrogels with good biocompatibility in a simple and efficient way are of practical significance for medical hydrogels.

This study aimed to covalently crosslink gelatin with PEG to form new composite hydrogels for controlled drug release. To this end, four-arm-PEG–succinimidyl glutarate (4-arm-PEG–SG) was first synthesized based on a commercially available 4-arm-PEG–OH and subsequently crosslinked with gelatin in situ to form gelatin–PEG hydrogels with various solid contents and crosslinking degrees. The corresponding dried gels were obtained using a freeze-drying technique. The 4-arm-PEG–SG was characterized using ^1^H Nuclear Magnetic Resonance (^1^H NMR) and Fourier Transform Infrared (FT–IR) spectroscopy, and the dried gels were characterized using FT–IR and a Scanning Electron Microscope (SEM). The effects of the solid content and crosslinking degree on the thermal properties, mechanical properties, swelling behavior, degradation performance, and drug release characteristics of the hydrogels were thoroughly investigated. Additionally, the biocompatibility of the hydrogels was evaluated by testing their cytotoxicity.

## 2. Materials and Methods

### 2.1. Chemicals

4-arm-PEG–OH (*M*_n_ = 10 kDa) was obtained from Sigma-Aldrich and vacuum-dehydrated at 70 °C for 3 h. Gelatin (cowhide, *M*_n_ = 150 kDa,) with –NH_2_ content of 5.14 × 10^−4^ mol g^−1^ was provided by National Pharmaceutical Chemical Reagent (Tianjin, China). Glutaric anhydride (GA, 99%), 4-dimethylaminopyridine (DMAP, 95%), *N*-hydroxysuccinimide (NHS, 95%), triethylamine (TEA, 99%), and 1-ethyl-(3-dimethylaminopropyl) carbodiimide (EDC, 97%) were purchased from Macklin (Shanghai, China). CFD (99.5%) was supplied by Shandong Tianming Pharmaceutical Co., Ltd. (Jinan, China). Fresh rabbit blood was sourced from Success Bio-Tech Co., Ltd. (Jinan, China). All other reagents were of analytical grade.

### 2.2. Experimental

#### 2.2.1. Synthesis of 4-arm-PEG–SG

**Figure 1:**
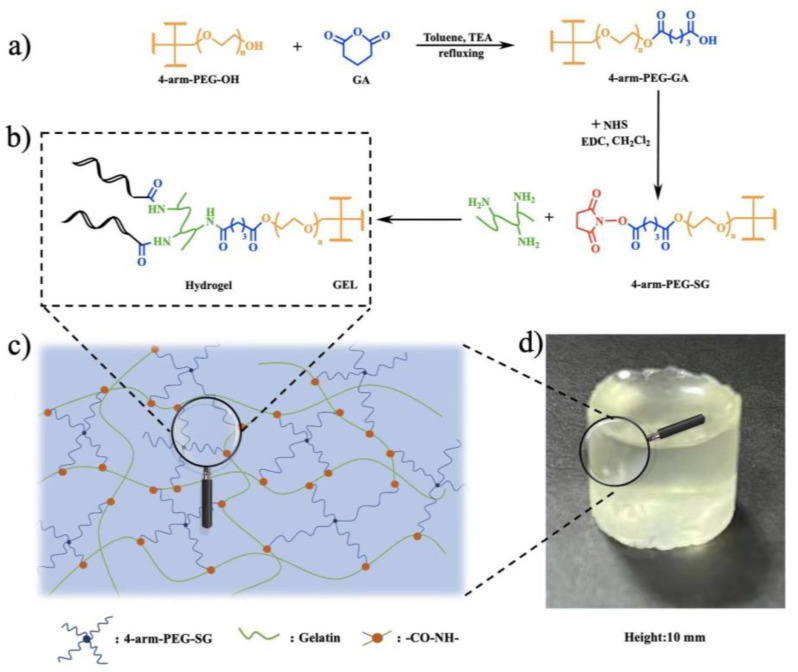
(**a**) Synthesis of 4-arm-PEG–SG; (**b**) preparation of S_n_N_m_ hydrogel; (**c**) possible crosslinking mechanism of hydrogel; and (**d**) optical image of hydrogel.

According to the reported method with minor modifications [24], 4-arm-PEG–SG was synthesized through a two-step esterification process, as shown in Figure 1a,b.

(a) 4-arm-PEG–OH (20.0 g), GA (2.75 g), and TEA (3.5 mL) were dissolved in 150 mL of toluene and refluxed for 24 h. After cooling the mixture to room temperature, the toluene was removed under reduced pressure. Deionized water (~100 mL) was added, and the solution was filtered. The filtrate was extracted with CH_2_Cl_2_ (40 × 30 × 30 mL), and the organic layers were dried by anhydrous Na_2_SO_4_. After the dried solution was concentrated to ~30 mL, cold diethyl ether (~200 mL, 0 °C) was poured into the solution under vigorous stirring. The resulting mixture was filtered to yield a pale-yellow crude product, which was further precipitated twice using the same method and vacuum-dried at room temperature to obtain 4-arm-PEG–glutarate acid (4-arm-PEG–GA) as a white powder. The yield was 78.3%.
(b) 4-arm-PEG–GA (15.0 g) and NHS (2.7 g) were dissolved in CH_2_Cl_2_ (200 mL) to obtain a homogenous solution. CH_2_Cl_2_ solution of EDC (35 mL, 0.06 g/mL) was then added dropwise into the solution under stirring. The reaction was performed at room temperature for 24 h. Subsequently, the mixture was filtered, and the filtrate was concentrated to ~30 mL. The cold diethyl ether (~250 mL, 0 °C) was added to the system under vigorous stirring to produce the crude product, which then underwent precipitation twice using the same method. After vacuum drying at room temperature, 4-arm-PEG–SG, as a white powder, was obtained. The yield was determined to be 85.9%.

#### 2.2.2. Preparation of Gelatin–PEG Crosslinked Hydrogels

The preparation process and crosslinking diagram of the hydrogels are illustrated in Figure 1b,c, respectively. The solid content of gelatin in the hydrogels was set at 1.5%, and the group molar ratios of –SG:–NH_2_ were set at 1:2, 1:1, and 2:1. 4-arm-PEG–SG and gelatin were dissolved in PBS with pH values of 4.0 and 9.0 at room temperature, respectively. The solutions were individually loaded into the tubes of a dual-chamber syringe equipped with a spiral-type nozzle. Subsequently, they were extruded and allowed to stand at ambient temperature for 5~10 min to form hydrogels (Figure 1d). The hydrogels were named as S_n_N_m_–I, where n:m denotes the molar ratio of –SG:–NH_2_. The –SG:–NH_2_ was fixed at 1:1, and hydrogels with solid contents of 2.5% and 3.5% were prepared using the same method, which were denoted as S_1_N_1_–II and S_1_N_1_–III, respectively. The hydrogels were then freeze-dried to obtain the corresponding dried gels, named as DS_n_N_m_.

### 2.3. Testing and Characterization

^1^H NMR spectra were recorded on an AVANCE II 400 MHz spectrometer (Bruker, Germany) with CDCl_3_ as the solvent and TMS as an internal standard. ^1^H NMR spectra were acquired using Bruker pulse program with the following settings: flip angle of 90°; relaxation delay (d1) = 20 s, size of fid (TD) = 65,536, number of scans (NS) = 64, spectral width (SW) = 20.0255 ppm, acquisition time (AQ) = 4.0894 s, requested probe temperature (TE) = 298.0 K. Data acquisition and processing were performed using MestReNova 9.0 (Mestrelab Research, S.L., Santiago de Compostela, Spain). FT–IR spectra were obtained on an ALPHA II infrared spectrometer (Bruker, Germany) equipped with an ATR accessory with 4 cm^−1^ resolution in the 4000 to 400 cm^−1^ absorbance range. The quantity of the sample and KBr in the pellets was about 2 mg/200 mg for all the studied samples. Three recordings were performed for each sample after successive milling. The sectional micro-morphologies of the lyophilized dry gel were observed by SEM (SU–8010, Hitachi, Japan). The samples were sputter-coated with gold before observation. The thermal stability and transition properties of the dry gel were assessed on a 2050 TGA (TGA, New Castle, DE, USA) and a 2910 DSC (Thermal, New Castle, DE, USA) with heating rates of 20 °C/min and 10 °C/min, respectively. The DSC curves were recorded from the second heating, and all tests were performed under a nitrogen atmosphere.

Swelling properties: The swelling properties were evaluated by measuring the water absorption rate of the samples [36]. The hydrogel was cut into a cylindrical shape with a cross-sectional diameter of 15 mm and a height of 5 mm, and the original weight was recorded as G_0_. The sample was immersed in deionized water at 37 °C. At specified intervals, the sample was removed and weighed as G_b_. The swelling rate (SR) was calculated using Formula (1).

(1) $$SR(\%)=\frac{G_{b}-G_{0}}{G_{0}}\times 100$$

Compression properties: The hydrogel sample was fabricated into a cylindrical shape (diameter: 15 mm; height: 10 mm) and was compressed on a CT3 Texture Analyzer (Brookfield, Middleboro, MA, USA) equipped with a TA10A probe. The compression rate and maximum compression strain were set as 0.01 mm/s and 90%, respectively. The test was performed at room temperature.

Degradability: The degradability of the hydrogels was assessed by measuring their mass loss rate in a PBS solution (pH 7.4). Samples that had reached swelling equilibrium were placed in a wet tea bag, and the overall mass was denoted as m_0_. The tea bags were subsequently immersed in PBS and incubated at 37 ± 0.1 °C. At specified time intervals, the tea bags were fetched out, left hanging for 3 min, and weighed as m_n_. The mass loss rate (MLR) for degradation was calculated according to Formula (2).

(2) $$MLR\left(\%\right)=\frac{m_{0}-m_{n}}{m_{0}}\times 100$$

Drug-release behavior: The sustained drug-release behavior of drug-loaded hydrogels in PBS (pH 7.4) was investigated using CFD as the model drug. The drug-loaded hydrogels were prepared by incorporating CFD into the 4-arm-PEG–SG and gelatin solution, followed by mixing and extruding the mixture through a dual-chamber syringe. The cylindrical drug-loaded hydrogels (diameter: 15 mm; height: 5 mm) with CFD of ~10 mg were immersed in PBS (10 mL) and incubated at 37 °C. At predetermined time intervals, the supernatant (1 mL) was withdrawn and diluted to 10 mL with fresh PBS. The absorbance at 205 nm was measured using UV–visible spectrophotometry. The released drug was calculated based on a standard curve, and the cumulative released drug was determined using Formula (3).

(3) $$CRD(\%)=\frac{c_{n}v_{n}v_{10}}{v_{1}\left(m_{0}-\frac{c_{n-1\times }v_{n-1\times }v_{10}}{v_{1}}\right)}\times 100$$

where *c*_n_ and *v*_n_ are the concentration of CFD and the volume of the solvent in the nth dilution solution, respectively; *v*_10_ is the volume of PBS (10 mL); *v*_1_ is the volume of the supernatant withdrawn (1 mL); and m_0_ is the total amount of CFD in the hydrogel.

Cell morphology: The cylindrical hydrogels with a diameter of 15 mm and a height of 2 mm were first immersed in 75% ethanol solution for 30 min, then rinsed three times with PBS after removal and sterilized under UV light for 30 min. The sterilized hydrogels were placed in a 24-well plate which contained 1.0 mL of culture medium, and cultured mouse fibroblasts (L929, ~10^4^ cells/well) were introduced. The multi-well plate was subsequently incubated at 37 °C in 5% CO_2_. After 72 h, the multi-well plate was removed, and the cell morphology on the hydrogel surface was observed using an inverted fluorescence microscope (XSP–15C, Shanghai Batuo Instrument, Shanghai, China).

Cytotoxicity: Cytotoxicity was assessed using the MTT assay, as reported in the previous literature [37,38]. Typically, sterilized samples were immersed in 2.5 mL of DMEM medium supplemented with 10% PBS. Following a 24 h extraction period at 37 ± 0.5 °C, the extract was filtered with a 0.22 μm filter, and the filtrate was diluted with 2.5 mL of fresh medium. A diluted solution (100 μL) was transferred to a 24-well plate containing L929 cells (~10^5^ cells/well). The cells cultured in pure medium served as the control. After 72 h of incubation at 37 °C in 5% CO_2_, cell viability was determined using the MTT assay. The optical density (OD) was measured at 570 nm with a 24-well plate reader, and the cell survival rate (CSR) was calculated according to Formula (4).

(4) $$CSR(\%)=\frac{A_{s}-A_{b}}{A_{c}-A_{b}}\times 100$$

where A_s_, A_b_, and A_c_ are the absorbance values of the sample, blank, and control groups, respectively. CSR ≥ 75% (class 0 or 1) indicated excellent cell compatibility.

Cell live/dead assay: After 72 h of culture as in the method in cytotoxicity, the culture medium was sucked out, and 250 μL of Calcein AM/PI staining solution was added to each well. The plate was then incubated in the dark for 30 min at 5% CO_2_ and 37 °C. The live/dead cells were observed under an inverted fluorescence microscope. Calcein AM emits green fluorescence (E_x_/E_m_ = 494/517 nm) for live cells, while PI emits red fluorescence (E_x_/E_m_ = 535/617 nm) for dead cells.

## 3. Results and Discussion

### 3.1. Synthesis of 4-arm-PEG–SG

**Figure 2:**
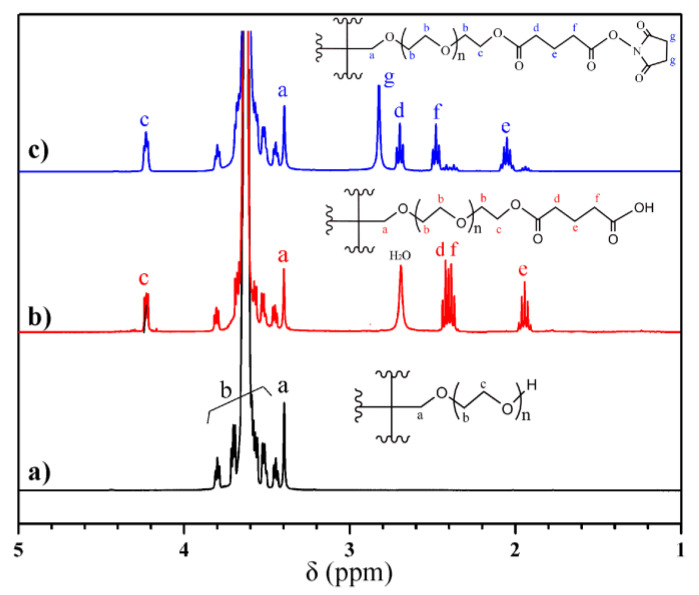
^1^H NMR spectra for (**a**) 4-arm-PEG–OH, (**b**) 4-arm-PEG–GA, and (**c**) 4-arm-PEG–SG with CDCl_3_ as solvent.

To achieve fast crosslinking between PEG and gelatin at ambient temperature and atmospheric pressure, the PEG needs to be modified, and thus the terminal –OH was activated by a two-step modification. The acylation reaction between 4-arm-PEG–OH and GA produced the carboxylic-acid-terminal 4-arm-PEG–GA, which then reacted with NHS to form activated ester, i.e., 4-arm-PEG–SG. The resultant 4-arm-PEG–SG can form hydrogels in the presence of gelatin. Figure 2 presents the ^1^H NMR spectra of 4-arm-PEG–OH, 4-arm-PEG–GA, and 4-arm-PEG–SG. The spectrum of 4-arm-PEG–OH (Figure 2a) exhibited no impurity peaks, and the distribution and positions of the proton peaks were consistent with the anticipated structure, indicating a high purity. Based on the ratio of the peak area of the repeating unit –O–CH_2_–CH_2_– (δ 3.42–3.80 ppm) to C–CH_2_–O (δ 3.38 ppm), the molecular weight was calculated to be ~10.5 kDa, which was consistent with the theoretical molecular weight [39]. The esterification of the terminal –OH of 4-arm-PEG–OH resulted in the formation of 4-arm-PEG–GA. The –CH_2_– proton peak of the terminal repeating unit –O–CH_2_–CH_2_– appeared at δ 4.22 ppm, and other proton peaks, as indicated in the ^1^H NMR spectrum (Figure 2b), were consistent with the expected structure. The absence of the –COOH proton peak in the spectrum was attributed to active hydrogen exchange with water in the sample (H_2_O: δ ~2.69 ppm). The substitution degree of the terminal –OH could be calculated using the integral area ratio of the peaks. S_e_:S_d,f_:S_a_ = 1.07:2.03:1.00 ≈ 1:2:1 means a substitution degree of ~100%. The ^1^H NMR spectrum of 4-arm-PEG–SG is shown in Figure 2c, and the proton peak positions are denoted in the figure. The degree of terminal group substitution was calculated from the area ratio of the newly appeared peak (e, f, d, g) to the –CH_2_– peak in the core (a). To avoid interference from the water proton peak (δ 2.5–3.0 ppm), the integral area of peak e was utilized for the accurate calculation. The total degree of terminal group substitution was found to be S_e_:S_a_ = 0.92:1.02 = 90.2%. In addition, the area ratio of S_g_ to S_e_ was approximately 2:1, indicating the absence of residual NHS in the product. No other impurity peaks were observed, suggesting the high purity of the 4-arm-PEG–SG.

### 3.2. Synthesis of Gelatin–PEG Hydrogels

The gelatin–PEG hydrogels were synthesized by the crosslinking of PEG with gelatin (Figure 1b,c). Hybrid hydrogels can be formulated into various forms, such as cylinders (Figure 1d). The resultant hydrogels were freeze-dried to obtain the corresponding dried gels (DS_n_N_m_). In order to be more intuitive, the compositions of the hydrogels are listed in Table 1.

**Figure 3:**
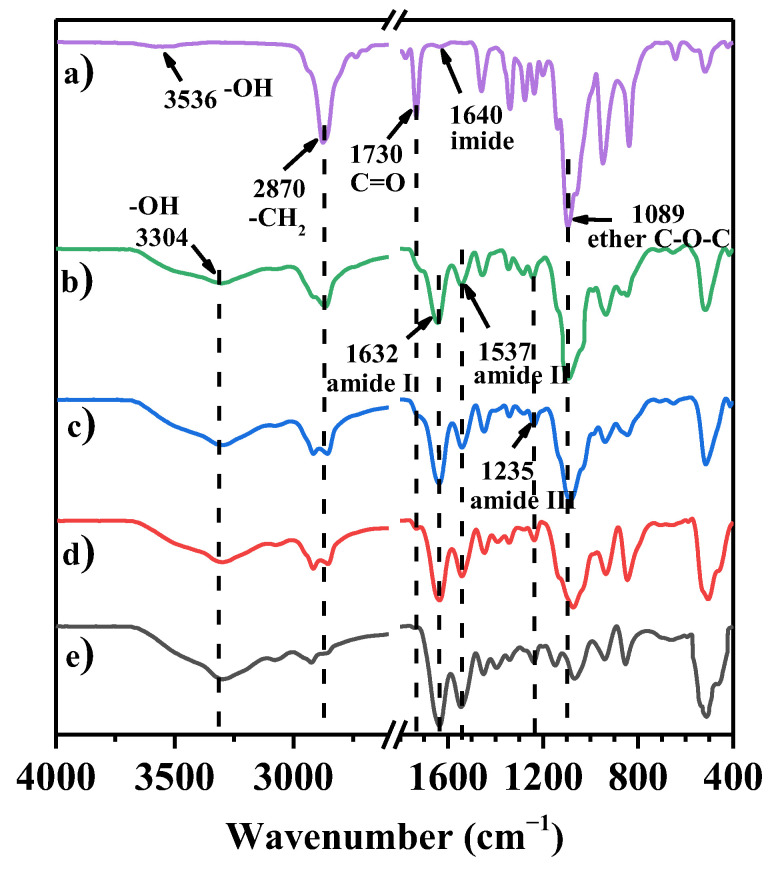
FT–IR spectra of (**a**) 4-arm-PEG–SG, (**b**) DS_2_N_1_–I, (**c**) DS_1_N_1_–I, (**d**) DS_1_N_2_–I, and (**e**) gelatin.

The FT–IR spectra of 4-arm-PEG–SG, DS_n_N_m_–I, and gelatin are displayed in Figure 3. The absorption peaks corresponding to the imide (1640 cm^−1^) and carbonyl groups of ester groups (~1730 cm^−1^) [40] in 4-arm-PEG–SG disappeared completely in the spectra of DS_n_N_m_–I, which indicates the successful esterification between the succinimide of the 4-arm-PEG–SG and the –NH_2_ of the gelatin. The amide I band of the newly formed amide groups overlapped with the amide I band of the gelatin (~1632 cm^−1^). In addition, compared to the gelatin, the amide II band (1537 cm^−1^) and amide III band (1235 cm^−1^) had no significant changes. In the FT–IR spectra of DS_n_N_m_–I (Figure 3b–d), the characteristic peaks of 4-arm-PEG–SG, such as –CH_2_– (2870 cm^−1^) and ether C–O–C (1089 cm^−1^), show increasing intensity with increasing PEG content, which is in accordance with the expected result. Meanwhile, the peak intensities of –OH (3304 cm^–1^) and the amide I, II, and III bands also increased with the increase in gelatin content. Thus, the FT–IR spectra confirmed the successful crosslinking between 4-arm-PEG–SG and gelatin.

### 3.3. Microscopic Morphology

**Figure 4:**
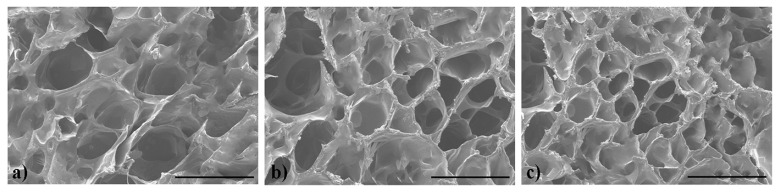
SEM images of (**a**) DS_2_N_1_–I, (**b**) DS_1_N_2_–I, and (**c**) DS_1_N_1_–I. (scale bar: 200 μm).

Figure 4 shows the SEM images of the dry gels. It can be observed that all the DS_n_N_m_-I exhibited a honeycomb-like structure with good interconnectivity [41], and the average pore size is: DS_2_N_1_-I ≈ DS_1_N_2_-I > DS_1_N_1_-I. The DS_1_N_1_-I has the smallest pore, but the most rigid structure, which could be ascribed to the equivalent crosslinking of 4-arm-PEG–SG and gelatin (–SG:–NH_2_ = 1:1). In other words, the crosslinking degree of DS_n_N_m_-I is: DS_1_N_1_-I > DS_2_N_1_-I ≈ DS_1_N_2_-I. The high porosity can enhance the contact area between the dry gel and water molecules, facilitating water storage within the pores. In addition, the dense pore wall can help to distribute forces when external pressure is applied, thereby maintaining the shape of the hydrogel. The hydrogels with high crosslinking degrees generally have dense three-dimensional network structures and improved mechanical properties [42]. Therefore, the porous microstructure meant good water absorbency, which was closely related to their swelling, degradation, and mechanical properties. Consequently, the performance of the hydrogels could be controlled by regulating their crosslinking degree. Furthermore, the hydrogels can be regenerated by immersing the dry gels in water.

### 3.4. Thermogravimetric Analysis (TGA)

**Figure 5:**
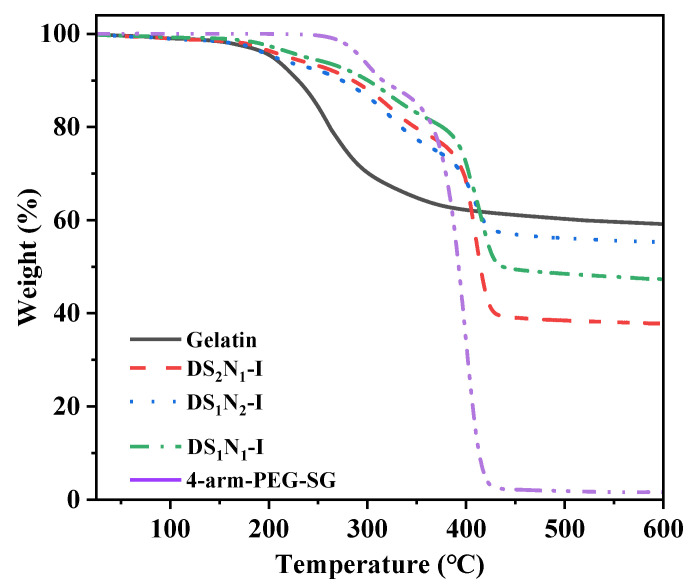
TGA curves of 4-arm-PEG–SG, DS_n_N_n_–I, and gelatin.

The TGA curves of the DS_n_N_m_–I samples (DS_1_N_1_–II and –III possess the same chemical composition and similar TGA curves as DS_1_N_1_–I) are illustrated in Figure 5, and characteristic values for the TGA curves are summarized in Table 2. It can be found that the weight loss of the dry gels was less than 2% at temperatures ~100 °C, which was attributed to the evaporation of the small molecular substances and residual moisture in the samples. The temperatures at 5% weight loss (T_5%_) for gelatin, DS_1_N_2_–I, DS_2_N_1_–I, DS_1_N_1_–I, and 4-arm-PEG–SG were 193.9, 202.5, 211.9, 220.1, and 296.7 °C, respectively, which demonstrated a gradually increasing trend in thermal stability. This phenomenon was ascribed to two factors. On the one hand, the crosslinking restricts the movement of the gelatin molecular chains, which need more energy for thermal decomposition, thereby resulting in a higher T_5%_. Therefore, DS_1_N_1_–I, with the highest crosslinking degree in the three gel samples, exhibited the highest T_5%_. On the other hand, the T_5%_ of 4-arm-PEG–SG was approximately 300 °C. Increasing the content of 4-arm-PEG–SG can enhance the thermal stability of DS_n_N_m_–I, resulting in a higher T_5%_ of DS_2_N_1_–I than DS_1_N_2_–I when their crosslinking degrees are similar. Pure gelatin exhibited a residual mass (W_r_) of ~60% at the end of the test (600 °C), indicating its superior thermal stability [43]. The W_r_ values for DS_2_N_1_–I, DS_1_N_1_–I, and DS_1_N_2_–I were 36.5%, 46.8%, and 55.3%, respectively. This tendency was consistent with increased gelatin content. The thermal weight loss of gelatin can be divided into two stages, ranging from 200 to 400 °C, which are initially decomposing into polypeptides and amino acids [44], followed by further thermal decomposition of these pyrolysis products. The thermal weight loss of the DS_n_N_m_–I was mainly categorized into three distinct stages. The slight mass loss at the first stage, with a maximum decomposition temperature (T_1_) of ~190 °C, was primarily attributed to the release of small molecules from the samples. The second stage, at T_2_ of ~270 °C, was due to the thermal decomposition of peptide bonds in the gelatin chain and ester bonds in 4-arm-PEG–SG. The third stage, with T_3_ at ~420 °C, displayed a major mass loss, which was due to the thermal decomposition of ether bonds in the PEG chain segments [29]. Overall, the incorporation of the crosslinking agent 4-arm-PEG–SG significantly enhanced the thermal stability of the gelatin. The T_5%_ values exceeded 200 °C, indicating that these materials were suitable for high-temperature sterilization.

### 3.5. Differential Scanning Calorimetry (DSC) Analysis

**Figure 6:**
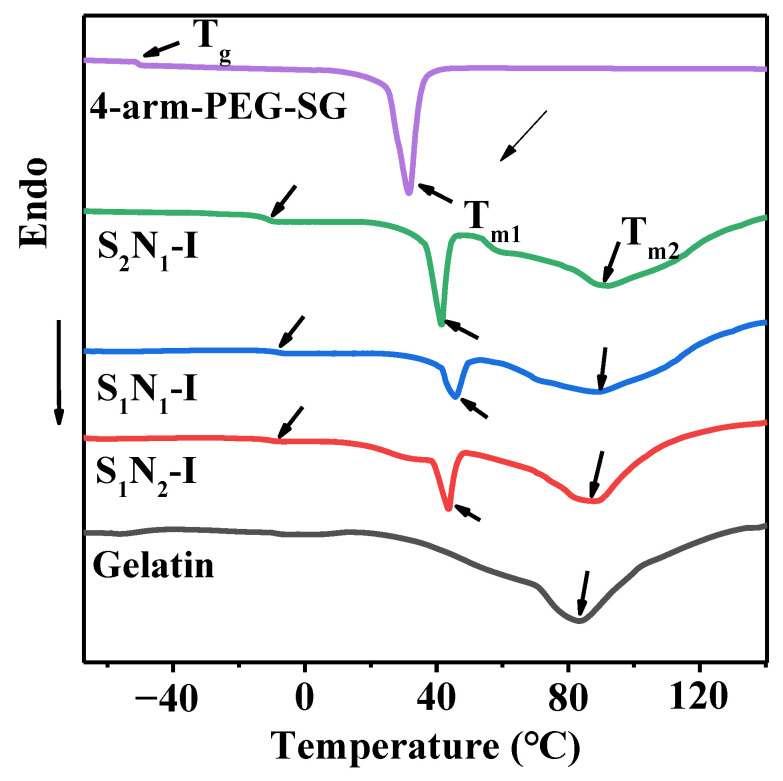
DSC curves of 4-arm-PEG–SG, DS_n_N_n_–I, and gelatin.

The DSC curves of 4-arm-PEG–SG, DS_n_N_m_–I, and gelatin are shown in Figure 6, and the characteristic values derived from these curves are listed in Table 3. As noted in the literature [45], no distinct glass transition temperature (T_g_) was observed in the curve of the gelatin. The T_g_ values for DS_1_N_2_–I, DS_1_N_1_–I, DS_2_N_1_–I, and 4-arm-PEG–SG were measured to be −10.6, −8.5, −11.2, and −49.6 °C, respectively. Crosslinking significantly led to a substantial reduction in the mobility of the molecular chains, resulting in higher T_g_ values for DS_n_N_m_–I than for 4-arm-PEG–SG [46]. The slight difference in T_g_ among the DS_n_N_m_–I samples could be attributed to two primary factors: one is that the high crosslinking degree produces strong intermolecular forces, which improve T_g_ value, and the other is that the high content of PEG, composed of soft segments, can lead to low T_g_ value, such as in DS_2_N_1_–I. Notably, the T_g_ values of all the DS_n_N_m_–I samples are below room temperature, indicating that the molecular chains of the hydrogels were in a highly elastic state at the application temperatures. Additionally, the gelatin exhibited a broad crystallization peak with a melting temperature (T_m2_) of ~80 °C, while the T_m1_ of 4-arm-PEG–SG was observed at 41.7 °C, indicating that the gelatin and 4-arm-PEG–SG possessed a certain degree of crystallinity [47]. DS_n_N_m_–I displayed a melting endothermic peak with T_m1_ at ~43 °C, which corresponded to the crystal melting peak of the PEG segment. The enthalpy (ΔH_1_) associated with the melting peak was inversely correlated with the crosslinking degree. The crosslinking restricts the free movement of molecular chains, thereby diminishing their crystallization capacity. For example, DS_1_N_1_–I, with the highest crosslinking degree among the DS_n_N_m_–I samples, demonstrated a crystal melting peak with the lowest enthalpy change. Another factor that influences the ΔH value is PEG content: high PEG content contributes to crystallization and results in a large ΔH value. When the crosslinking degree was the same, DS_2_N_1_–I, which contained a higher PEG content, possessed a larger ΔH_1_ value than DS_1_N_2_–I. DS_n_N_m_–I presented a broad T_m2_ peak at ~80 °C, which belonged to the crystal melting peak of the gelatin component. The corresponding ΔH_2_ values followed the same relationship with ΔH_1_.

### 3.6. Swelling Performance

**Figure 7:**
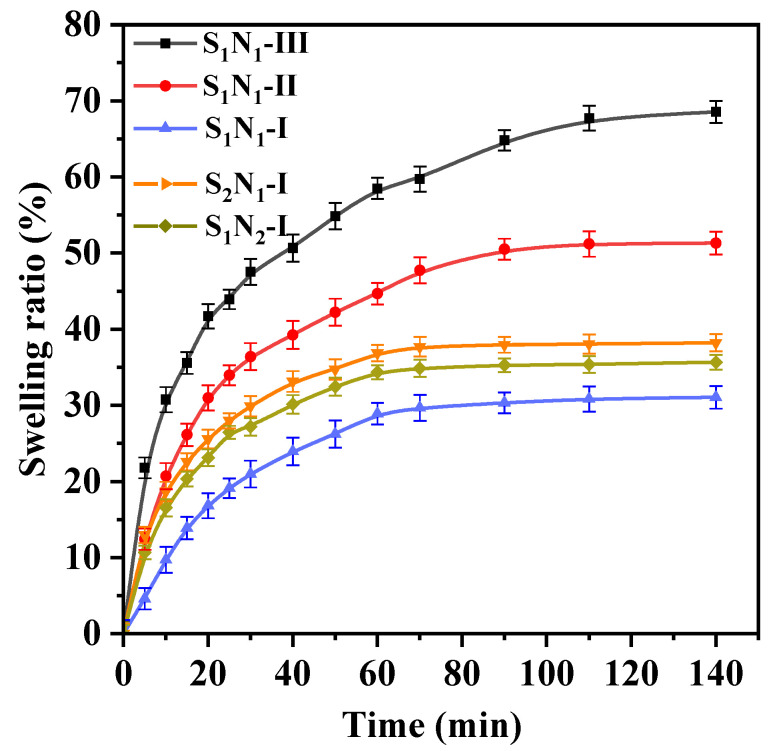
Swelling ratio of S_n_N_m_ hydrogels in water at 37 °C.

The swelling ratios (SRs) of the hydrogels S_n_N_m_ in deionized water at 37 °C over time are illustrated in Figure 7. The SR of S_n_N_m_ presented a rapid increase in the initial stage, followed by a gradual increase. The S_n_N_m_–I samples have the same solid content, and their SR increased rapidly within the first 30 min and subsequently reached swelling equilibrium at ~80 min. The equilibrium swelling ratios (ESR) for S_1_N_1_–I, S_1_N_2_–I, and S_2_N_1_–I were 30.65%, 31.45%, and 37.95%, respectively. The ESR of the samples tended to decrease with the increase in crosslinking degree [48]. The ESR of S_2_N_1_–I was slightly higher than that of S_1_N_2_–I. This could be due to the increased content of hydrophilic PEG, which enhanced the hydrophilicity of hydrogels. For the samples that had the same crosslinking degree (S_1_N_1_–I, –II, and –III), the SR exhibited an initial rapid increase and gradually reached swelling equilibrium at ~120 min. With the increase in solid content (i.e., from S_1_N_1_–I to S_1_N_1_–II, and further to S_1_N_1_–III), the ESR values presented a significant increasing trend from 30.65%, to 51.32% and further to 68.54%. A high solid content means more components of hydrophilic gelatin and PEG, thereby leading to an enhancement in ESR. Furthermore, the high solid content of hydrogels also contributes to their stability, which could prevent dissolution before achieving equilibrium swelling. Therefore, the swelling capacity of the S_n_N_m_ could be effectively controlled by changing the crosslinking degree and/or solid content.

### 3.7. Compression Performance

**Figure 8:**
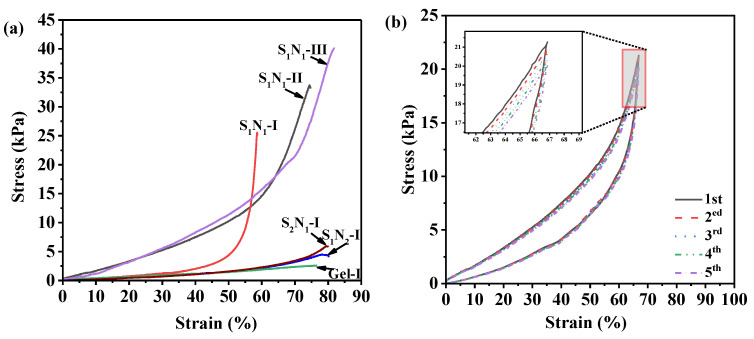
(**a**) Compressive stress–strain curve of S_n_N_m_ hydrogels and (**b**) cyclic compressive stress–strain curves of S_1_N_1_–II.

An important requirement for the practical application of hydrogels is their ability to withstand external pressures without failure. Hydrogels with high compression resistance capacity can extend their application range. The compression stress–strain curves of the S_n_N_m_ are displayed in Figure 8a. Gel–I, prepared from 1.5% pure gelatin, serves as a control. Gel–I exhibited weaker mechanical strength, indicating that the pure gelatin cannot be applied directly. When the compression deformation (ε) exceeds 20%, the stress–strain curve of S_1_N_1_–I shows a significant difference compared to S_2_N_1_–I and S_1_N_2_–I, which required larger stress to achieve the same ε. The maximum compressive strength (σ_m_) of S_1_N_1_–I could reach 25.03 kPa, whereas the σ_m_ of S_2_N_1_–I and S_1_N_2_–I were only 5.89 and 4.46 kPa, respectively. This difference should be attributed to the fact that a denser three-dimensional network formed under a high crosslinking degree reduces the mobility of the molecular chains, resulting in requiring more energy to disrupt its structure [49]. S_1_N_2_–I and S_2_N_1_–I, having similar crosslinking degrees, displayed analogous compression stress–strain curves and maximum compressive deformation (ε_m_) of ~80%, while the ε_m_ of S_1_N_1_–I was only 58.37%. As the solid content increased (S_1_N_1_–I~–III), both the σ_m_ and ε_m_ values were enhanced greatly. S_1_N_1_–II and S_1_N_1_–III achieved σ_m_ of 33.17 kPa and 40.08 kPa, and ε_m_ of 74% and 82%, respectively. The reason is that high solid content could lead to a decrease in the diameter of the pores and an increase in pore density, as observed by SEM. Although the mechanical properties of the prepared hydrogels are improved by the increase in solid content, an excessive solid content can increase the brittleness and decrease the toughness of the hydrogels. Therefore, an appropriate crosslinking degree and a solid content of S_n_N_m_ hydrogels are needed to achieve good compressive performance and toughness.

For further investigation of the compression performance, S_1_N_1_–II with good comprehensive performance was adopted as the representative sample to perform the cyclic compression tests. The ε was set to 90% of ε_m_, and the cyclic compressive stress–strain curves are shown in Figure 8b. As expected, the σ_m_ gradually decreased with increasing cycles. However, after five cycles, the S_1_N_1_–II exhibited nearly overlapping stress–strain curves, and the σ_m_ remained above 95% of the initial value, indicating superior anti-fatigue performance compared to the inelastic cellulose hydrogel [50]. This good cyclic compression performance could be attributed to the amide and hydrogen bonds and the crosslinked network structure within the hydrogels.

### 3.8. In Vitro Degradation Performance

**Figure 9:**
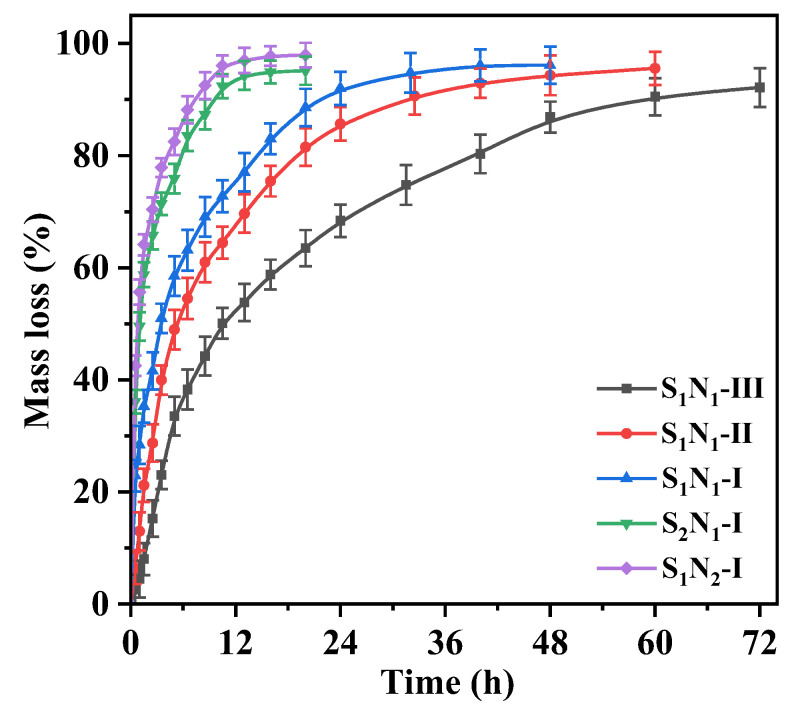
Mass loss curves of S_n_N_m_ hydrogels against the degradation time in PBS (pH 7.4) at 37 °C.

Degradability is a crucial requirement for drug-loaded hydrogels. The degradation performances of the S_n_N_m_ hydrogels were assessed by measuring their mass loss over time in PBS (pH 7.4, 37 °C), and the degradation curves are shown in Figure 9. After 12 h of degradation, the mass losses for S_1_N_2_–I, S_2_N_1_–I, S_1_N_1_–I, S_1_N_1_–II, and S_1_N_1_–III were recorded as 96.99%, 94.32%, 77.05%, 64.45%, and 53.80%, respectively. This indicated that S_1_N_2_–I and S_2_N_1_–I were nearly completely degraded, while S_1_N_1_–I, S_1_N_1_–II, and S_1_N_1_–III were relatively stable. The degradation rate was primarily affected by the solid content and crosslinking degree: high crosslinking degree and/or solid content contribute to the improvement of biostability. S_1_N_1_–III, which had the highest crosslinking degree and solid content in the samples, required 72 h to achieve 90% mass loss. The results demonstrated that the prepared hydrogels were degradable, and the complete degradation time could be controlled between 12 and 72 h by adjusting the crosslinking degree and solid content.

### 3.9. Drug Release Performance

**Figure 10:**
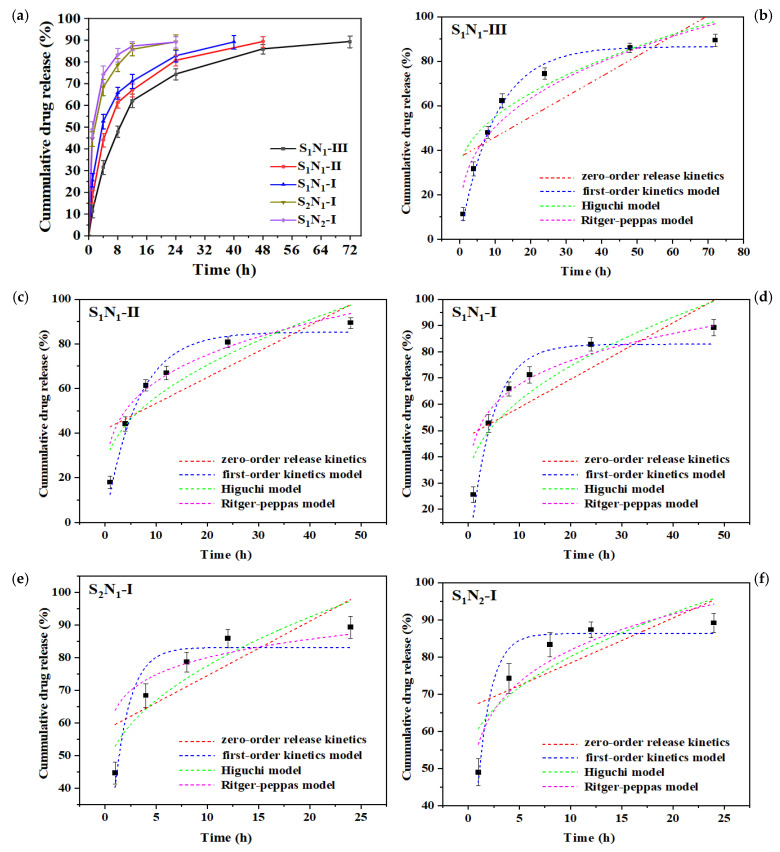
(**a**) Drug release profiles from S_n_N_m_ hydrogels in PBS (pH 7.4) at 37 °C; release kinetics of (**b**) S_1_N_1_-III, (**c**) S_1_N_1_-II, (**d**) S_1_N_1_-I, (**e**) S_2_N_1_-I, and (**f**) S_1_N_2_-I.

Cefazedone sodium (CFD), a first-generation cephalosporin antibiotic, possesses strong antimicrobial activity against various Gram-positive bacteria [51]. It remains widely used for treating bacteria-caused systemic infections and prophylaxis before surgical procedures. Figure 10 illustrates the CFD release profiles of S_n_N_m_ hydrogels in PBS (pH 7.4) at 37 °C. It was found that all the hydrogels exhibited similar drug-release profiles, which could be divided into two distinct stages: initial burst release and subsequent sustained release. A high drug release rate was displayed in the initial release stage, which was due to the release of the drug located within the outer surface of the hydrogels. With the swelling of the hydrogels, the diffusion of the drugs in the superficial layer of the hydrogels accelerated the release rate. Subsequently, the drug release rate decreased gradually, until the hydrogels were largely degraded and ~90% of the drug was released. In this stage, the drug release rate was mainly controlled by the degradation rate. The S_1_N_2_–I and S_2_N_1_–I samples exhibited similar release profiles, with 80% of the drug released within 8 h and complete drug release (~90%) after 24 h. In contrast, the sustained release duration for S_1_N_1_–I, S_1_N_1_–II, and S_1_N_1_–III was prolonged: they released 70–80% of the drug after 24 h, subsequently entering the sustained release phase, with 90% CFD being released after 40, 48, and 72 h, respectively. The increase in crosslinking degree and solid content in hydrogels could extend their drug release time, and the drug-release profiles were consistent with their degradation curves. Santi groups also found a similar phenomenon of degradation-controlled drug release in PEG-based hydrogel [52].

In order to detect the influence of network parameters (crosslinking degree and solid content) on the release kinetics of the S_n_N_m_ hydrogels, the release profiles were approximated using several mathematical models, including zero-order, first-order, Higuchi and Ritger–Peppas, to determine the release behavior of CFD from the hydrogels. The data analysis is described in the supporting information. It can be seen that the CFD release data showed the best fit to the first-order equations in all the cases (the release parameters are shown in Figures S1–S5), suggesting that the transport rate of CFD within the matrix of hydrogels was proportional to the concentration of the drug where it was located. Thereby, we can see that the network parameters of hydrogels, including crosslinking degree and solid content, played an important role in the sustained drug release. It seems that a high crosslinking degree is a prerequisite for hydrogels for controlled drug release, as the hydrogels with a low crosslinking degree degrade too quickly. As expected, the release rate of the hydrogels can be controlled by changing their solid content. From the perspective of practical application, the mechanical properties, thermal stability, and chemical stability of hydrogels also need to be considered. Thus, a high crosslinking degree and high solid content in the gelatin–PEG hydrogels are advantageous for sustained CFD release.

### 3.10. Cytocompatibility and Cell Viability

**Figure 11:**
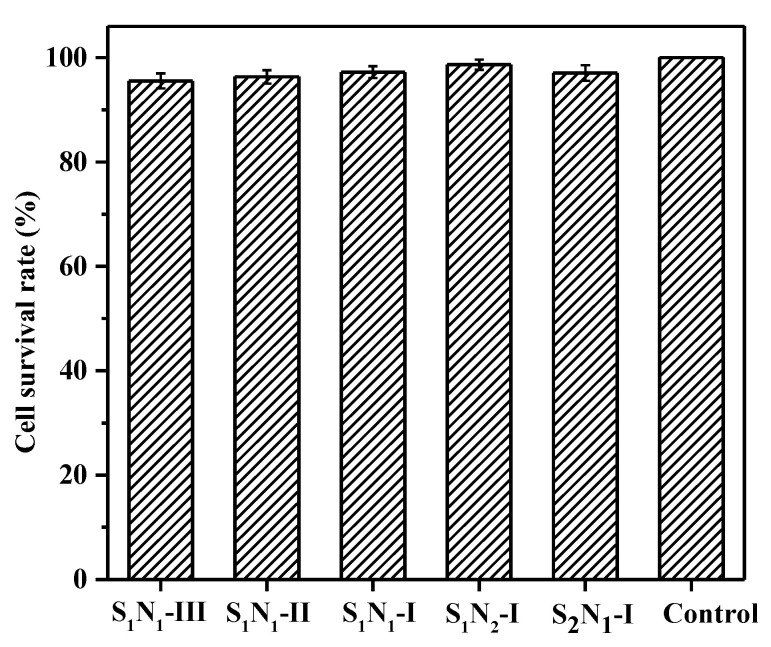
Cell survival rate in S_n_N_m_ hydrogel extracts using MTT at 37 °C for 72 h.

**Figure 12:**
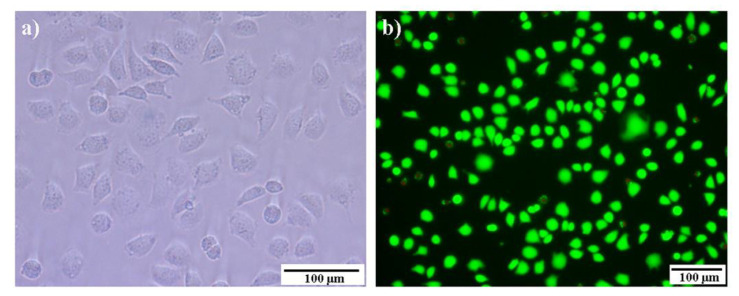
(**a**) Cell morphologies on S_1_N_1_–I hydrogel surface and (**b**) live/dead cells in S_1_N_1_–I extracts (37 °C, 72 h).

Biocompatibility is also a critical factor for the evaluation and selection of medical materials. In this section, cytocompatibility was used as a primary method for assessing biocompatibility. L929 cells and the MTT assay were adopted to evaluate the cytotoxicity of the prepared hydrogels. The cell survival rates (CSRs) of S_n_N_m_ hydrogels after 72 h of incubation are shown in Figure 11. All the samples exhibited >95% CSR values, meaning they achieved desirable results in comparison with the control. The high cell viability should be attributed to the introduction of gelatin, which was a bioderived polypeptide and possessed excellent biocompatibility [53]. Similar results have been reported in PEG–gelatin hydrogels based on the thiol-ene click reaction [34]. Based on the standard of ISO 10993-6:2016 [54], the cytotoxicity was grade 1, which indicated that the prepared hydrogels were nontoxic and met the requirements of in vivo materials. The morphologies of the cells on the S_1_N_1_–I hydrogel surface were observed, and the micrographs are shown in Figure 12a. It is evident that most of the cells were in close contact with one another and displayed spread morphologies, indicating a favorable growth condition [55]. In addition, the cell viability in the S_1_N_1_–I hydrogel extracts was observed using a live/dead assay (Figure 12b). It was found that a large number of cells remained viable, and almost no dead cells were observed. Similar results were also observed for other hydrogels, like S_2_N_1_–I and S_1_N_2_–I (Figures S6 and S7). These tests indicated that the hydrogels exhibited minimal cytotoxicity and excellent cytocompatibility.

## 4. Conclusions

In summary, a series of novel hybrid gelatin–PEG hydrogels (S_n_N_m_) with different solid contents and crosslinking degrees were prepared through in situ crosslinking between 4-arm-PEG–SG and gelatin. The successful crosslinking of gelatin–PEG hydrogels was confirmed by ^1^H NMR and FT–IR spectra. The resultant hydrogels were characterized by SEM, TGA, and DSC, and their properties, including thermal stability, mechanical stability, compressive resistance, and degradation, were investigated. The effects of solid content and crosslinking degrees on the properties of S_n_N_m_ hydrogels were intensively detected. The results indicated that the increase in the crosslinking degree and solid content enhanced the thermal stability and compressive resistance, and they extended the degradation time of the hydrogels. The drug release capacity was mainly controlled by the degradation properties of the hydrogels, and the sustained drug-release time could be modulated by adjusting the crosslinking degree or solid content. The cytotoxicity of the hydrogels was evaluated using the MTT assay, and the CSR values exceeding 95% demonstrated excellent cytocompatibility. This study offers new insights and methodologies for the development of hydrogels as biomedical materials.

## Figures and Tables

**Table 1:** The compositions of the hydrogels.

Samples	–SG:–NH_2_	Solid Content (%)	State
S_1_N_2_–I	1:2	1.5	hydrogels
S_1_N_1_–I	1:1	1.5	hydrogels
S_2_N_1_–I	2:1	1.5	hydrogels
S_1_N_1_–II	1:1	2.5	hydrogels
S_1_N_1_–III	1:1	3.5	hydrogels
DS_n_N_m_	n:m	100	dried gels

**Table 2:** Characteristic values of 4-arm-PEG–SG, DS_n_N_n_–I, and gelatin from TGA curves.

Samples	T_5%_/°C	T_1_/°C	T_2_/°C	T_3_/°C	W_r_/%
gelatin	193.9	189.3	272.1	–	59.1
DS_2_N_1_–I	211.9	192.1	324.0	415.3	36.5
DS_1_N_2_–I	202.5	193.9	326.1	418.6	55.3
DS_1_N_1_–I	220.1	197.9	329.8	423.0	46.8
4-arm-PEG–SG	296.7	–	303.5	404.4	1.6

**Table 3:** Characteristic parameters of 4-arm-PEG–SG, DS_n_N_n_–I, and gelatin from DSC curves.

Samples	T_g_ (°C)	T_m1_ (°C)	T_m2_ (°C)	Δ*H*_1_ (J/g)	Δ*H*_2_ (J/g)
4-arm-PEG–SG	−49.6	41.7	–	29.07	–
DS_1_N_2_–I	−10.6	43.6	88.2	18.59	43.57
DS_1_N_1_–I	−8.5	44.6	90.3	13.80	37.85
DS_2_N_1_–I	−11.2	42.8	86.8	23.14	50.95
gelatin	–	–	82.8	–	72.86

## Data Availability

The data are contained within the article.

## Supplementary Materials

The following supporting information can be downloaded at: https://www.mdpi.com/article/10.3390/molecules29204952/s1. Figure S1: The release kinetics of S_1_N_1_-III; Figure S2: The release kinetics of S_1_N_1_-II; Figure S3: The release kinetics of S_1_N_1_-I. Figure S4: The release kinetics of S_2_N_1_-I; Figure S5: The release kinetics of S_1_N_2_-I; Figure S6: Left: Cell morphologies on S_2_N_1_-I hydrogel surface; right: live/dead cells in S_2_N_1_-I extracts (37 °C, 72 h); Figure S7: Left: Cell morphologies on S_1_N_2_–I hydrogel surface; right: live/dead cells in S_1_N_2_–I extracts (37 °C, 72 h).

## References

[1] Hu X.H., Zhang C.Y., Xiong Y.S., Ma S.M., Sun C., Xu W.L. (2024). A Review of Recent Advances in Drug Loading, Mathematical Modeling and Applications of Hydrogel Drug Delivery Systems. J. Mater. Sci..

[2] Sun Z., Song C., Wang C., Hu Y., Wu J. (2020). Hydrogel-Based Controlled Drug Delivery for Cancer Treatment: A review. Mol. Pharmacol..

[3] Ali L., Ahmad M., Usman M., Yousuf M. (2014). Controlled Release of Highly Water-Soluble Antidepressant from Hybrid Copolymer Poly Vinyl Alcohol Hydrogels. Polym. Bull..

[4] Lee K., Mooney D.J. (2001). Hydrogels for Tissue Engineering. Chem. Rev..

[5] Wang C.G., Surat’man N.E.B., Chang J.J., Ong Z.L., Li B.F., Fan X.T., Loh X.J., Li Z.B. (2022). Polyelectrolyte Hydrogels for Tissue Engineering and Regenerative Medicine. Chem. Asian. J..

[6] Li L.L., Wang Y.Q., Pan L.J., Shi Y., Cheng W., Shi Y., Yu G.H. (2015). A Nanostructured Conductive Hydrogels-Based Biosensor Platform for Human Metabolite Detection. Nano Lett..

[7] Swilem A.E., Oyama T.G., Oyama K., Kimura A., Taguchi M. (2022). Development of Carboxymethyl Cellulose/Gelatin Hybrid Hydrogels via Radiation-Induced Cross-Linking as Novel Anti-Adhesion Barriers. Polym. Degrad. Stabil..

[8] Kasai R.D., Radhika D., Archana S., Shanavaz H., Koutavarapu R., Lee D.Y., Shim J. (2023). A Review on Hydrogels Classification and Recent Developments in Biomedical Applications. Int. J. Polym. Mater. Polym. Biomater..

[9] Shen Z., Zhang C., Wang T., Xu J. (2023). Advances in Functional Hydrogel Wound Dressings: A Review. Polymers.

[10] Teng Y., Li S., Tang H., Tao X., Fan Y., Huang Y. (2023). Medical Applications of Hydrogels in Skin Infections: A Review. Infect. Drug Resist..

[11] Gionet-Gonzales M., Casella A., Diloretto D., Ginnell C., Griffin K.H., Bigot A., Leach J.K. (2021). Sulfated Alginate Hydrogels Prolong the Therapeutic Potential of MSC Spheroids by Sequestering the Secretome. Adv. Healthc. Mater..

[12] Ickenstein L.M., Garidel P. (2018). Hydrogel Formulations for Biologicals: Current Spotlight from A Commercial Perspective. Ther. Deliv..

[13] Tan B., Huang L., Wu Y., Liao J. (2021). Advances and Trends of Hydrogel Therapy Platform in Localized Tumor Treatment: A Review. J. Biomed. Mater. Res. A.

[14] Thang N.H., Chien T.B., Cuong D.X. (2023). Polymer–Based Hydrogels Applied in Drug Delivery: An Overview. Gels.

[15] Cascone S., Lamberti G. (2020). Hydrogel–Based Commercial Products for Biomedical Applications: A Review. Int. J. Pharm..

[16] Bordbar-Khiabani A., Gasik M. (2022). Smart Hydrogels for Advanced Drug Delivery Systems. Int. J. Mol. Sci..

[17] Wang Z., Ye Q., Yu S., Akhavan B. (2023). Poly Ethylene Glycol (PEG)-Based Hydrogels for Drug Delivery in Cancer Therapy: A Comprehensive Review. Adv. Healthc. Mater..

[18] Cui R., Wu Q., Wang J., Zheng X., Ou R., Xu Y., Qu S., Li D. (2021). Hydrogel-By-Design: Smart Delivery System for Cancer Immunotherapy. Front. Bioeng. Biotech..

[19] Naahidi S., Jafari M., Logan M., Wang Y., Yuan Y., Bae H., Dixon B., Chen P. (2017). Biocompatibility of Hydrogel–Based Scaffolds for Tissue Engineering Applications. Biotechnol. Adv..

[20] Xue X., Hu Y., Deng Y., Su J. (2021). Recent Advances in Design of Functional Biocompatible Hydrogels for Bone Tissue Engineering. Adv. Funct. Mater..

[21] Vanti G., Wang M., Bergonzi M.C., Liu Z., Bilia A.R. (2020). Hydroxypropyl Methylcellulose Hydrogel of Berberine Chloride–Loaded Escinosomes: Dermal Absorption and Biocompatibility. Int. J. Biol. Macromol..

[22] Zhu J. (2010). Bioactive Modification of Poly(Ethylene Glycol) Hydrogels for Tissue Engineering. Biomaterials.

[23] Grover G.N., Lam J., Nguyen T.H., Segura T., Maynard H.D. (2012). Biocompatible Hydrogels by Oxime Click Chemistry. Biomacromolecules.

[24] Li J., Kao W.J. (2003). Synthesis of Polyethylene Glycol (PEG) Derivatives and PEGylated-Peptide Biopolymer Conjugates. Biomacromolecules.

[25] Kim I., Choi J.S., Lee S., Byeon H.J., Lee E.S., Shin B.S., Choi H.G., Lee K.C., Youn Y.S. (2015). In Situ Facile-Forming PEG Cross-Linked Albumin Hydrogels Loaded with an Apoptotic TRAIL Protein. J. Control. Release.

[26] Sargeant T.D., Desai A.P., Banerjee S., Agawu A., Stopek J.B. (2012). An In Situ Forming Collagen–PEG Hydrogel for Tissue Regeneration. Acta Biomater..

[27] Chang C.W., Spreeuwel A.N., Zhang C., Varghese S. (2010). PEG/Clay Nanocomposite Hydrogel: A Mechanically Robust Tissue Engineering Scaffold. Soft. Matter..

[28] Kang J.I., Park K.M. (2021). Advances in Gelatin–Based Hydrogels for Wound Management. J. Mater. Chem. B.

[29] Yuan X., Zhu Z., Xia P., Wang Z., Zhao X., Jiang T., Gao Q., Xu J., Shan D., Guo B. (2023). Tough Gelatin Hydrogel for Tissue Engineering. Adv. Sci..

[30] Mohanto S., Narayana S., Merai K.P., Kumar J.A., Bhunia A., Hani U., Fatease A.A., Gowda B.H.J., Nag S., Ahmed M.G. (2023). Advancements in Gelatin–Based Hydrogel Systems for Biomedical Applications: A State-of-the-Art Review. Int. J. Biol. Macromol..

[31] Kommareddy S., Amiji M. (2007). Poly(Ethylene Glycol)–Modified Thiolated Gelatin Nanoparticles for Glutathione-Responsive Intracellular DNA Delivery. Nanomed. Nanotechnol..

[32] Kaul G., Amiji M. (2002). Long-Circulating Poly(Ethylene Glycol)-Modified Gelatin Nanoparticles for Intracellular Delivery. Pharm. Res..

[33] Kasper F.K., Jerkins E., Tanahashi K., Barry M.A., Tabata Y., Mikos A.G. (2006). Characterization of DNA Release from Composites of Oligo(Poly(Ethylene Glycol) Fumarate) and Cationized Gelatin Microspheres In Vitro. J. Biomed. Mater. Res. B.

[34] Dong Y., Sigen A., Rodrigues M., Li X., Kwon S., Kosaric N., Khong S., Gao Y., Wang W., Gurtner G.C. (2017). Injectable and Tunable Gelatin Hydrogels Enhance Stem Cell Retention and Improve Cutaneous Wound Healing. Adv. Funct. Mater..

[35] Liang J., Guo Z., Timmerman A., Grijpma D., Poot A. (2019). Enhanced mechanical and cell adhesive properties of photo-crosslinked PEG hydrogels by incorporation of gelatin in the networks. Biomed. Mater..

[36] Fumio U., Hiroshi Y., Kumiko N., Sachihiko N., Kenji S., Yasunori M. (1990). Swelling and Mechanical Properties of Poly(vinyl alcohol) Hydrogels. Int. J. Pharm..

[37] Lan X., Luo T., Zhong Z., Huang D., Liang C., Liu Y., Wang H., Tang Y. (2022). Green cross-linking of gelatin/tea polyphenol/ε-poly (L-lysine) electrospun nanofibrous membrane for edible and bioactive food packaging. Food Packag. Shelf. Life.

[38] Rodríguez-Rodríguez R., García-Carvajal Z., Jiménez-Palomar I., Jiménez-Avalos J., Espinosa-Andrews H. (2019). Development of gelatin/chitosan/PVA hydrogels: Thermal stability, water state, viscoelasticity, and cytotoxicity assays. J. Appl. Polym. Sci..

[39] Wu M., Yong Z. (1985). ^1^H–NMR Determine Molecular Weight of Polyether Polyols. Polym. Commun..

[40] Chen H., Fei F., Li X., Nie Z., Zhou D., Liu L., Zhang J., Zhang H., Fei Z. (2021). A Structure-Supporting, Self-Healing, and High Permeating Hydrogel Bioink for Establishment of Diverse Homogeneous Tissue-Like Constructs. Bioact. Mater..

[41] Zhang Y., Dong Q., Zhao X., Sun Y., Lin X., Zhang X., Wang T., Yang T., Jiang X., Li J. (2024). Honeycomb-Like Biomimetic Scaffold by Functionalized Antibacterial Hydrogel and Biodegradable Porous Mg Alloy for Osteochondral Regeneration. Front. Bioeng. Biotech..

[42] Yu Y., Kong N., Hou Z., Men L., Yang P., Wang Z. (2024). Sponge-Like Porous Polyvinyl Alcohol/Chitosan-Based Hydrogel with Integrated Cushioning, pH-Indicating and Antibacterial Functions. Int. J. Biol. Macromol..

[43] Hossan M.J., Gafur M.A., Kadir M.R., Karim M.M. (2014). Preparation and Characterization of Gelatin-Hydroxyapatite Composite for Bone Tissue Engineering. Int. J. Eng. Technol..

[44] Mahesh B., Kathyayani D., Gowda D.C., Sionkowska A., Ramakrishna S. (2022). Miscibility and Thermal Stability of Synthetic Glutamic Acid Comprising Polypeptide with Polyvinyl Alcohol: Fabrication of Nanofibrous Electrospun Membranes. Mater. Chem. Phys..

[45] Mukherjee I., Rosolen M. (2013). Thermal Transitions of Gelatin Evaluated Using DSC Sample Pans of Various Seal Integrities. J. Therm. Anal. Calorim..

[46] Yang B., Yin S., Bian X., Liu C., Liu X., Yan Y., Zhang C., Zhang H., Hou Z. (2022). Preparation and Properties of Monomethoxyl PolyEthylene Glycol Grafted O–Carboxymethyl Chitosan for Edible, Fresh–Keeping Packaging Materials. Food Packag. Shelf. Life.

[47] Banpean A., Sakurai S. (2021). Confined Crystallization of Poly(Ethylene Glycol) in Spherulites of Poly (L-Lactic Acid) in a PLLA/PEG Blend. Polymer.

[48] Bigi A., Cojazzi G., Panzavolta S., Roveri N., Rubini K. (2002). Stabilization of Gelatin Films by Crosslinking with Genipin. Biomaterials.

[49] Liu Z., Chen L., Qu L., Zhang R., Qin Z., Zhang H., Wei J., Xu J., Hou Z. (2024). Cross–Linked Poly(Ester Urethane)/Starch Composite Films with High Starch Content as Sustainable Food–Packaging Materials: Influence of Cross–Link Density. Int. J. Biol. Macromol..

[50] Gao X., Shi Z., Liu C., Yang G., Sevostianov I., Silberschmidt V.V. (2015). Inelastic Behaviour of Bacterial Cellulose Hydrogel: In Aqua Cyclic Tests. Polym. Test..

[51] Gao L., Zhu Y., Lyu Y., Hao F.L., Zhang P., Wei M.J. (2015). A Pharmacokinetic and Pharmacodynamic Study on Intravenous Cefazedone Sodium in Patients with Community-Acquired Pneumonia. Chin. Med. J..

[52] Ashley G.W., Henise J., Reid R., Sant D.V. (2013). Hydrogel Drug Delivery System with Predictable and Tunable Drug Release and Degradation Rates. Proc. Natl. Acad. Sci. USA.

[53] Xu X., Feng Q., Ma X., Deng Y., Zhang K., Ooi H.S., Yang B., Zhang Z., Feng B., Bian L. (2022). Dynamic gelatin-based hydrogels promote the proliferation and self-renewal of embryonic stem cells in long-term 3d culture. Biomaterials.

[54] (2016). Biological Evaluation of Medical Devices. Part 6: Tests for Local Effects after Implantation.

[55] Thomsa V., Jayabalan M. (2001). Studies on the Effect of Virtual Crosslinking on the Hydrolytic Stability of Novel Aliphatic Polyurethane Ureas for Blood Contact Applications. J. Biomed. Mater. Res..

