# Supplementary material for: Crosslinked Biodegradable Hybrid Hydrogels Based on Poly(ethylene glycol) and Gelatin for Drug Controlled Release
Source: Molecules. 2024 Oct 19;29(20):4952. doi: 10.3390/molecules29204952 (PMC11510199; doi:10.3390/molecules29204952)
Supplement: Supplementary file 1 [file molecules-29-04952-s001.zip › molecules-3250542-supplementary.pdf]

Crosslinked Biodegradable Hybrid Hydrogels Based on Poly(ethylene glycol) and Gelatin for Drug Controlled Release

Zhenzhen Zhao [1], Zihao Qin [2], Tianqing Zhao [3], Yuanyuan Li [1,*], Zhaosheng Hou [2,*], Hui Hu [3], Xiaofang Su [3] and Yanan Gao [3]

[1] School of Advanced Agricultural Science, Weifang University, Weifang 261061, China
[2] College of Chemistry, Chemical Engineering and Materials Science, Shandong Normal University, Jinan 250014, China
[3] Key Laboratory of Ministry of Education for Advanced Materials in Tropical Island Resources, Hainan University, Haikou 570228, China
* Correspondence: yylilove@126.com (Y.L.); houzs@sdnu.edu.cn (Z.H.)

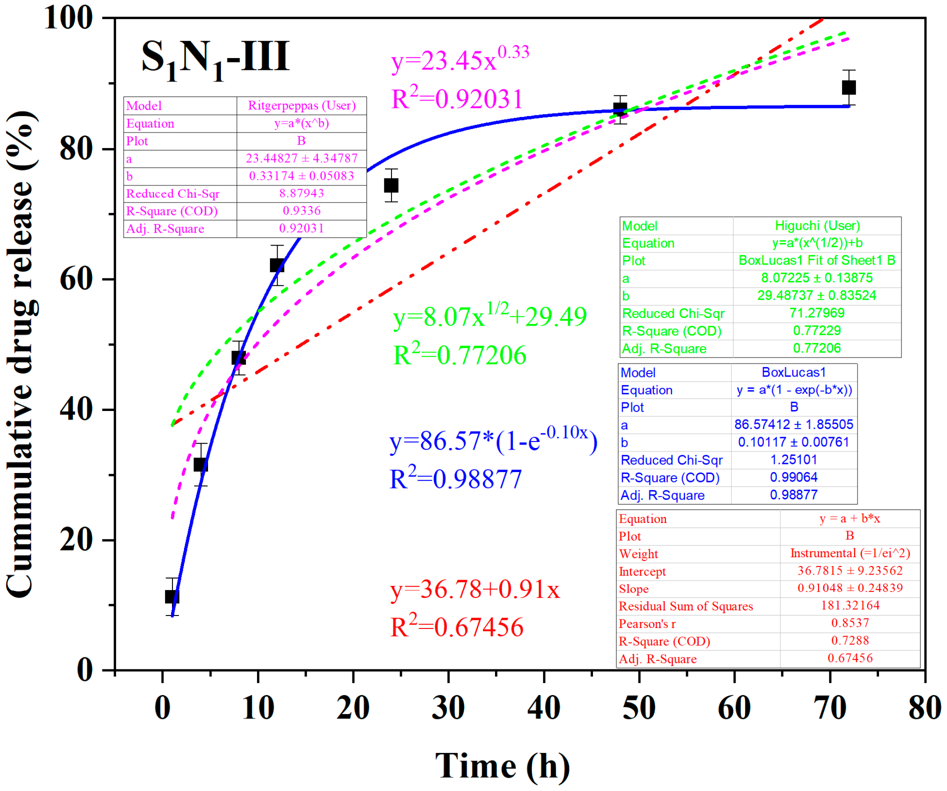

Figure S1. The release kinetics of S_1N_1-III.

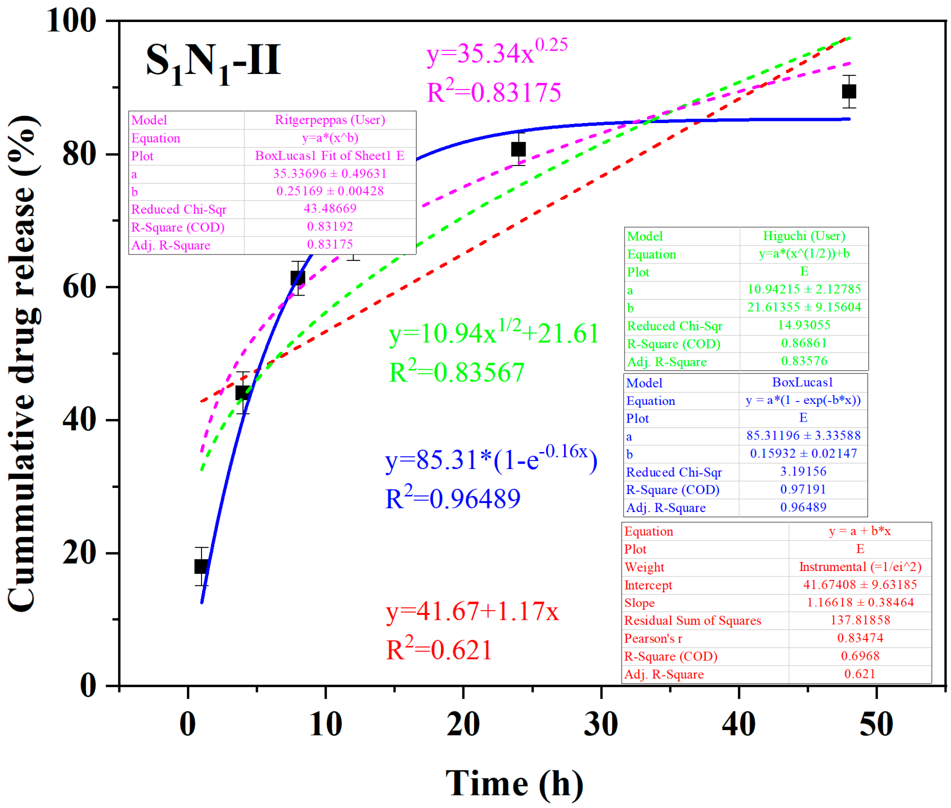

Figure S2. The release kinetics of S_1N_1-II.

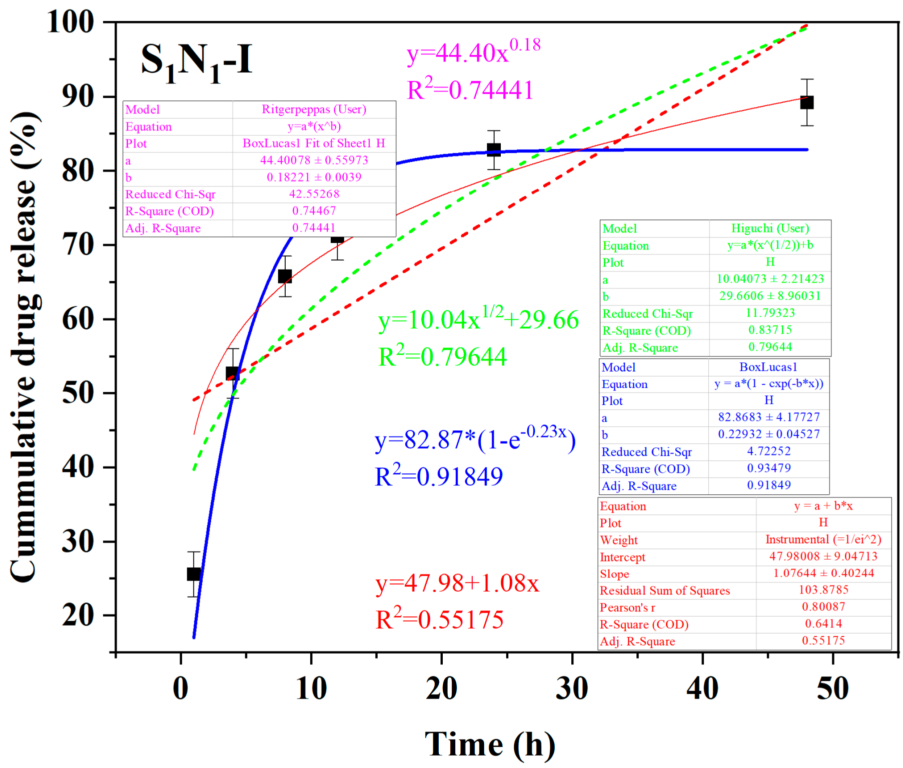

Figure S3. The release kinetics of S_1N_1-I.

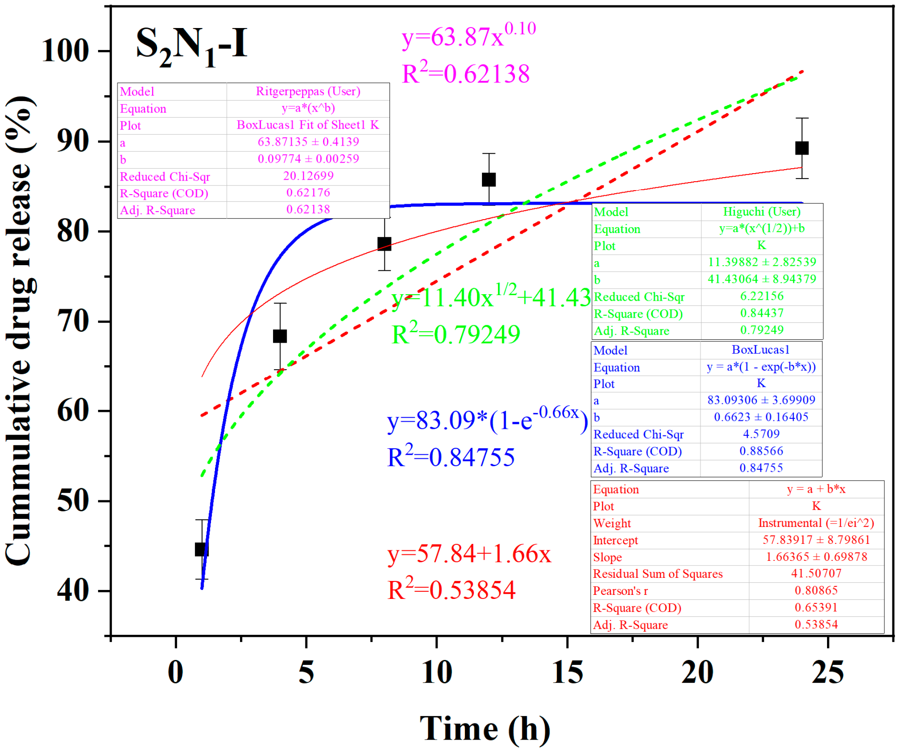

Figure S4. The release kinetics of S_2N_1-I.

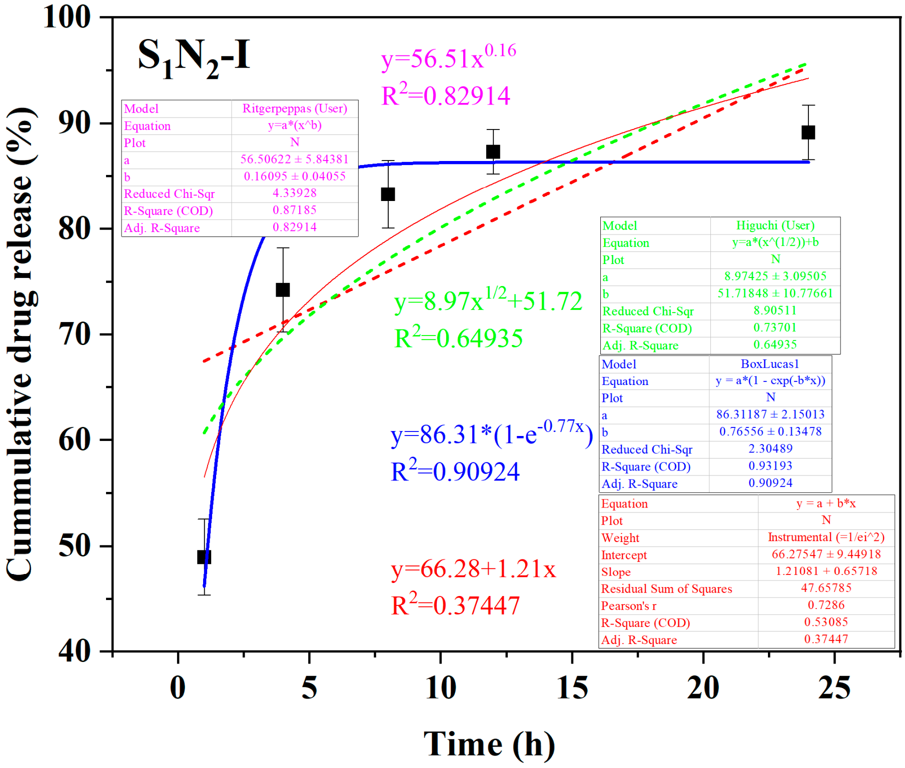

Figure S5. The release kinetics of S_1N_2-I.

Drug release kinetics

The probable mechanism of drug release from the hydrogels was investigated by analyzing the release data according to four basic empirical mathematical equations. The data were analyzed using OriginPro 7 (OriginLab Corporation).

Model 1: Zero-order kinetics

$$\frac{M_t}{M_\infty} = kt \quad \text{(S1)}$$

where Mt and M∞ are the cumulative release amount of spinosad at time t and at infinite time, respectively. k is the zero-order kinetic rate constant, and t is the release time.

Model 2: First-order kinetics

$$\ln(1 - \frac{M_t}{M_\infty}) = -kt \quad \text{(S2)}$$

where Mt and M∞ are the cumulative release amount of spinosad at time t and at infinite time, respectively. k is the first-order kinetic rate constant, and t is the release time.

Model 3: Higuchi equation that describes the Fickian diffusion of a drug:

$$\frac{M_t}{M_\infty} = kt^{1/2} \quad \text{(S3)}$$

where Mt and M∞ are the cumulative release amount of spinosad at time t and at infinite time, respectively. k is a kinetic constant, and t is the release time.

Model 4: Ritger–Peppas equation:

$$\frac{M_t}{M_\infty} = kt^n \quad \text{(S4)}$$

where Mt and M∞ are the cumulative release amount of spinosad at time t and at infinite time, respectively. k is a kinetic constant, and t is the release time. n is the diffusional exponent that can be related to the drug transport mechanism. For a thin hydrogel film, when $n \leq 0.45$, the drug release mechanism is Fickian diffusion, which occurs by the usual molecular diffusion of the drug due to a chemical potential gradient. When the value of n is between 0.45 and 0.89, anomalous transport (non-Fickian) is observed where both Fickian and relaxational phenomena contribute to the drug release. When $n > 0.89$, the drug release mechanism is case II transport.

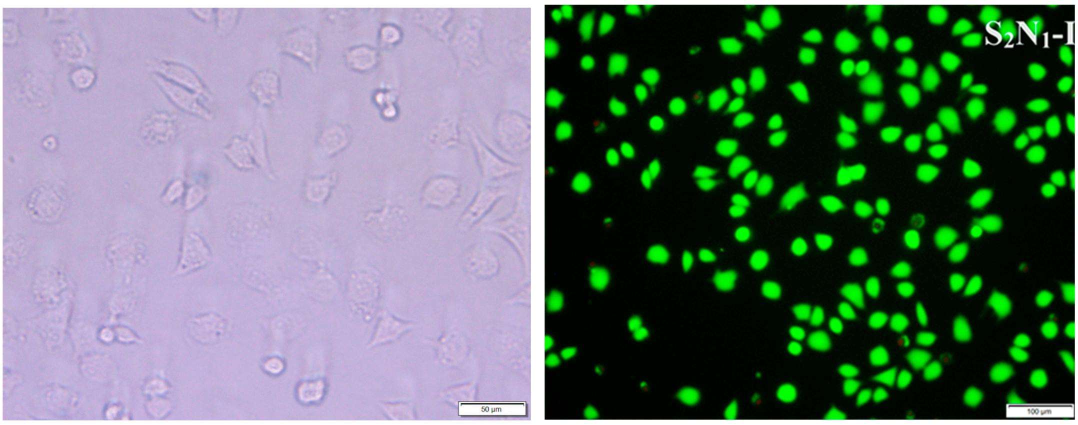

Figure S6. Left: Cell morphologies on S_2N_1−I hydrogel surface; Light: live/dead cells in S_2N_1−I extracts (37 ℃, 72 h).

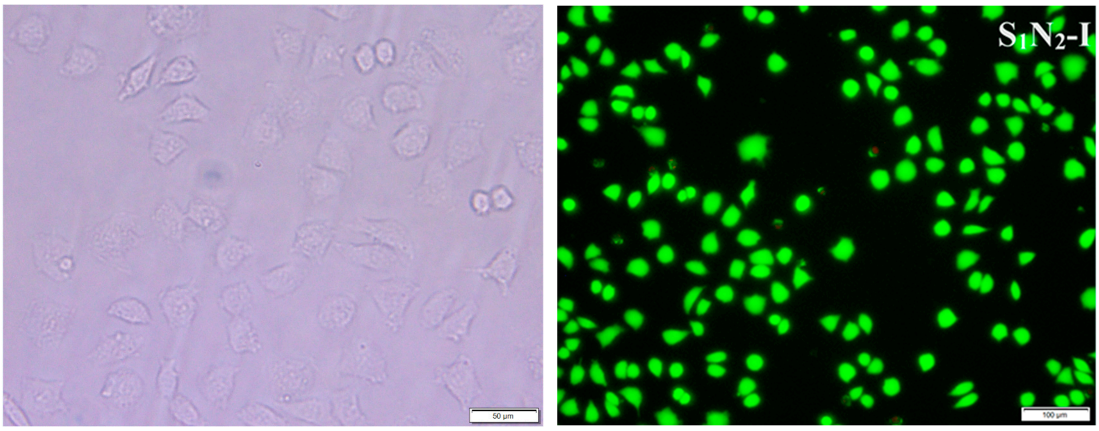

Figure S7. Left: Cell morphologies on S_1N_2−I hydrogel surface; Right: live/dead cells in S_1N_2−I extracts (37 ℃, 72 h).
